# Supplementary material for: Real-Time Fluorescence Visualization and Quantitation of Cell Growth and Death in Response to Treatment in 3D Collagen-Based Tumor Model
Source: Int J Mol Sci. 2022 Aug 9;23(16):8837. doi: 10.3390/ijms23168837 (PMC9408454; doi:10.3390/ijms23168837)
Supplement: Supplementary file 1 [file ijms-23-08837-s001.zip › ijms-1856730-supplementary.pdf]

Supplementary information for article

Real-time fluorescence visualization and quantitation of cell growth and death in response to treatment in 3D collagen-based tumor model

Ludmila M. Sencha, Olga E. Dobrynina, Anton D. Pospelov, Evgenii L. Guryev, Nina N. Peskova, Anna A. Brilkina, Elena I. Cherkasova and Irina V. Balalaeva

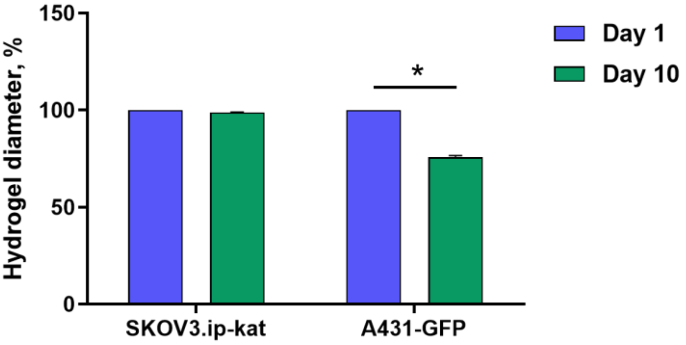

Figure S1. Collagen gel contraction with embedded SKOV3.ip-kat and A431-GFP cells on days 1 and 10 of cultivation. The data are presented as mean ± standard deviation. * – statistically significant difference between the variants (Shidak's test, $p < 0.05$).

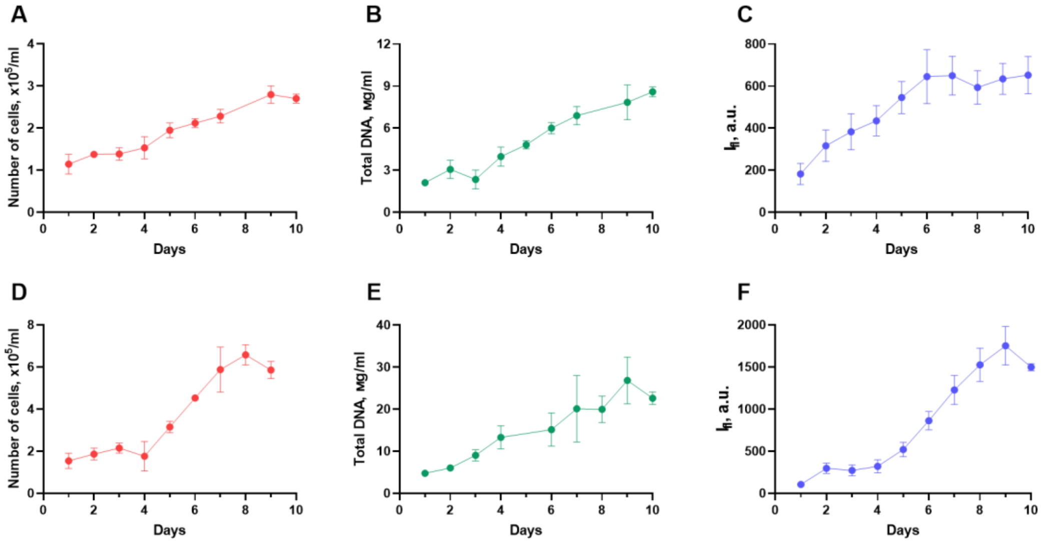

Figure S2. Growth curves of cell cultures SKOV3.ip(kat) (A, B, C) and A431(GFP) (D, E, F) plotted basing on the count of cells isolated from hydrogels (A, D); counting the content of total DNA (B, E); analysis of the fluorescence signal from hydrogels with cells (C, F). The data are presented as mean ± standard deviation.

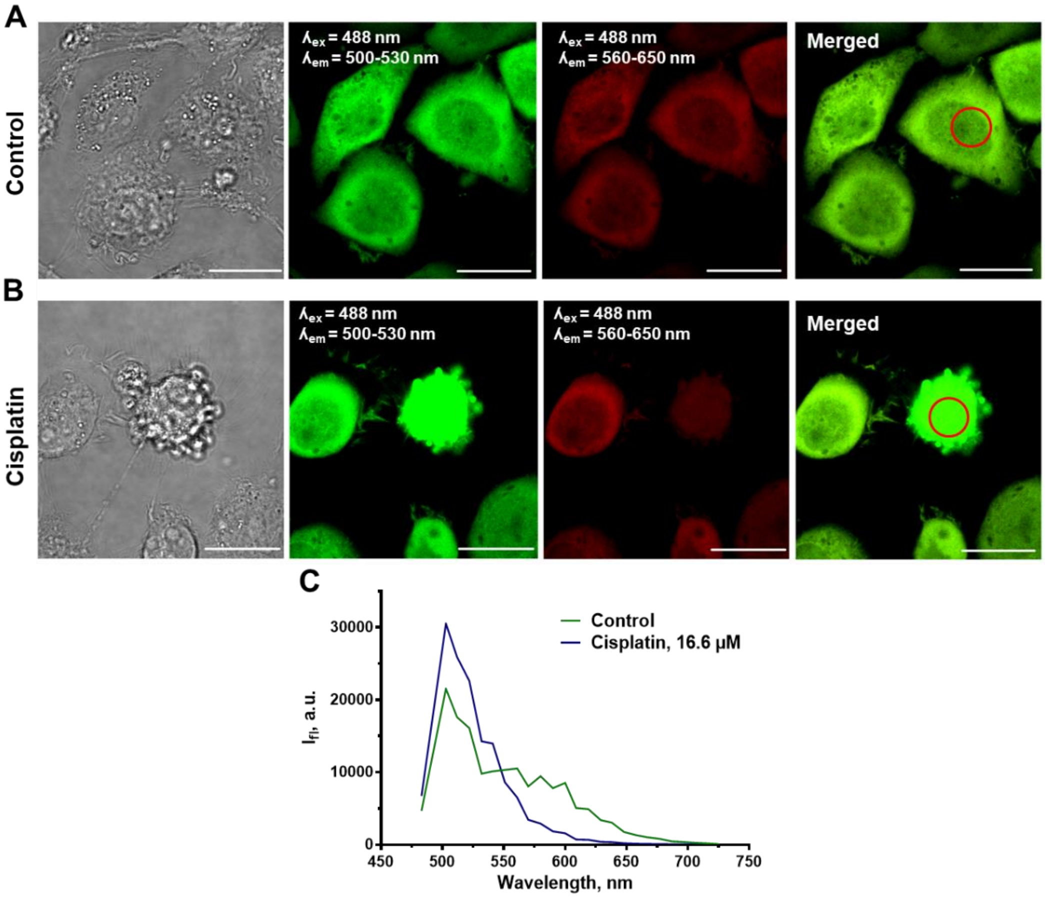

Figure S3. Test for functionality of the genetically encoded sensor Casper3-GR in cells of A431-Casper3GR line. (A, B) Images of A431-Casper3GR cells, obtained before (A) and 12 after the addition to culture medium of 16.6 μM cisplatin (B). The images were obtained using laser scanning confocal microscopy (AxioObzerver Z1 LSM710, Carl Zeiss, Garmany). The channel in transmitted light; fluorescence of the donor of the sensor (λ_{ex} 488 nm, λ_{em} 500-530 nm); fluorescence of the acceptor of the sensor under donor ixcitation (λ_{ex} 488 nm, λ_{em} 560-650 nm); and merged fluorescence images are presented. Bar, 20 μM. (C) The emission spectra of the sensor obtained in region of interests marked by red circles in A and B. The spectra were registered with 10 nm step

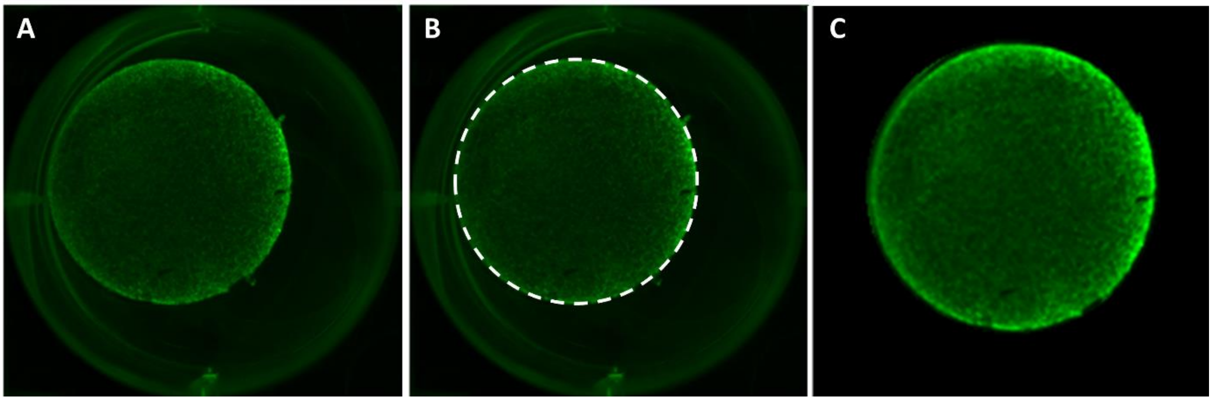

Figure S4. Illustration of the procedure for obtaining and analyzing fluorescent images of collagen hydrogels with embedded A431-GFP cells: obtaining images of gels in the wells of the plate (A), highlighting the gel area (dashed line) (B), calculating the integral fluorescence signal in the selected area (C).
